# Comparative Persistence of the TNF Antagonists in Rheumatoid Arthritis – A Population-Based Cohort Study

**DOI:** 10.1371/journal.pone.0105193

**Published:** 2014-08-20

**Authors:** Anat Fisher, Ken Bassett, James M. Wright, M. Alan Brookhart, Hugh Freeman, Colin R. Dormuth

**Affiliations:** 1 Therapeutics Initiative, University of British Columbia, Vancouver, Canada; 2 Department of Anesthesiology, Pharmacology & Therapeutics, University of British Columbia, Vancouver, Canada; 3 Department of Family Practice, University of British Columbia, Vancouver, Canada; 4 Department of Medicine, University of British Columbia, Vancouver, Canada; 5 Department of Epidemiology, Gillings School of Global Public Health, University of North Carolina, Chapel Hill, North Carolina, United States of America; University of Leuven, Rega Institute, Belgium

## Abstract

**Objective:**

To compare persistence with tumor necrosis factor alpha (TNF) antagonists among rheumatoid arthritis patients in British Columbia. Treatment persistence has been suggested as a proxy for real-world therapeutic benefit and harm of treatments for chronic non-curable diseases, including rheumatoid arthritis. We hypothesized that the different pharmacological characteristics of infliximab, adalimumab and etanercept cause statistically and clinically significant differences in persistence.

**Methods:**

We conducted a population-based cohort study using administrative health data from the Canadian province of British Columbia. The study cohort included rheumatoid arthritis patients who initiated the first course of a TNF antagonist between 2001 and 2008. Persistence was measured as the time between first dispensing to discontinuation. Drug discontinuation was defined as a drug-free interval of 180 days or switching to another TNF antagonist, anakinra, rituximab or abatacept. Persistence was estimated and compared using survival analysis.

**Results:**

The study cohort included 2,923 patients, 63% treated with etanercept. Median persistence in years (95% confidence interval) with infliximab was 3.7 (2.9–4.9), with adalimumab 3.3 (2.6–4.1) and with etanercept 3.8 (3.3–4.3). Similar risk of discontinuation was observed for the three drugs: the hazard ratio (95% confidence interval) was 0.98 (0.85–1.13) comparing infliximab with etanercept, 0.95 (0.78–1.15) comparing infliximab with adalimumab and 1.04 (0.88–1.22) comparing adalimumab with etanercept.

**Conclusions:**

Similar persistence was observed with infliximab, adalimumab and etanercept in rheumatoid arthritis patients during the first 9 years of use. If treatment persistence is a good proxy for the therapeutic benefit and harm of these drugs, then this finding suggests that the three drugs share an overall similar benefit-harm profile in rheumatoid arthritis patients.

## Introduction

The tumor necrosis factor alpha (TNF) antagonists are a relatively new class of drugs used to treat multiple inflammatory diseases, including rheumatoid arthritis (RA). These drugs bind and inactivate TNF, a cytokine that primarily acts as a proinflammation mediator and is thought to be important in the pathogenesis of RA [1].

Treatment with a TNF antagonist is not a cure [2] but rather reduces the symptoms of inflammation, possibly inducing remission and preventing long-term complications.

Randomized clinical trials (RCTs) are limited in their ability to provide evidence on the relative benefit and harm of TNF antagonists in real-world setting for several reasons. First, there are no head-to-head RCTs comparing two (or more) individual TNF antagonists. Multiple indirect comparisons have reported inconsistent estimates of relative efficacy and safety [3]–[9]. Second, the duration of RCTs is considerably shorter than the lifetime of the disease. Since RA is characterized by a variation in disease activity and deterioration over time, effects observed in a short-term trial may not be significant in the long-term and a drug which had no short-term effects may prevent complications or progression of the disease over the long-term [10], [11]. Last, RA patients who participate in RCTs designed to assess therapeutic benefit and harm of TNF antagonists are not representative of actual RA populations treated with TNF antagonists in routine clinical settings [12]–[15]. Furthermore, patients eligible to participate in RCTs had an improved response compared to ineligible patients [12]–[14], and therefore an overestimation of therapeutic benefit is likely.

Real-life drug persistence was suggested as a simple indirect approach for assessing long-term therapeutic benefit and harm [10]. This suggestion is based on the assumption that when using a drug that reduces symptoms and prevents complications (but does not cure the disease), patients persist with the treatment as long as they experience or perceive a benefit and they do not experience (or perceive) an unacceptable amount of harm. This theoretical perspective on persistence seems to hold for TNF antagonist drugs – the main reasons for discontinuing or switching these drugs in RA were decreased benefit (36–67% of the discontinuations) or perceived harm (30–58%) [16]–[21].

Estimates of comparative persistence with TNF antagonists in RA patients remain controversial and estimates could not be pooled due to differences in measures of persistence (e.g. median, mean, percentage of persistent patients after 1-year) [22], [23]. Hence, high quality research is warrant to provide evidence on comparative persistence of these drugs.

Each TNF antagonist has different pharmacological properties [24]–[26] and therefore substantive differences in therapeutic benefit and harm are expected. Since most discontinuations of these drugs are due to decreased benefit or increased harm, we hypothesized that differences in therapeutic effects would lead to differences in persistence between the TNF anatgonists in RA patients. The main objective of the current study was to compare persistence with infliximab, adalimumab and etanercept in RA in the Canadian province of British Columbia.

## Patients and Methods

Patients were identified using four British Columbia Ministry of Health administrative databases: PharmaNet (pharmacy dispensing data), Medical Service Plan (MSP) registration information (demographic data), MSP payment information (fee-for-service payments to physicians and alternative providers), and the Discharge Abstract Database (hospital separations). The databases were linked using a unique anonymized identifier. Follow-up data were available until December 31, 2009. The study protocol was approved by the Clinical Research Ethics Board of the University of British Columbia. Patient records and physician information were anonymized and de-identified prior to analysis.

The study cohort was identified based on (1) exposure to infliximab, adalimumab or etanercept and (2) diagnosis of RA. Exposure to TNF antagonist was defined as at least one recorded dispensing claim of infliximab, adalimumab or etanercept between March 2001 and December 31, 2008. The index date was the date of the first dispensing event. We defined a pre-study period of three years preceding the index date during which patients were required to have continuous provincial MSP coverage. A gap shorter than 30 days was not considered to be an interruption in MSP coverage. The pre-study period ensured a standard run-in period of at least three years without TNF antagonist exposure and a standard period during which diagnosis of RA was required.

RA patients were selected into the study cohort if they had two outpatient visits in physician clinics with a diagnosis code of RA (ICD-9 714.XX) at least 60 days apart, or at least one hospitalization with a recorded discharge diagnosis of RA in the pre-study period. This method was similar, though not identical, to criteria previously used in British Columbia data [27]. Patients entering the cohort were assigned to mutually exclusive exposure categories based on the first TNF antagonist they received (i.e., infliximab, adalimumab or etanercept). None of the patients was dispensed two or more different TNF antagonists at the index date. Patients were excluded if they were previously treated with anakinra, rituximab or abatacept, or if either sex or date of birth was not available. In addition, patients were excluded if they had a concurrent diagnosis of Crohn's disease (based on at least one outpatient or inpatient diagnosis code in the pre-study period) or if they were younger than 18 years at the index date (to remove patients with juvenile RA).

During the study period, clinical guidelines and the provincial drug coverage policy considered the drugs comparable [28], [29]; nevertheless, the choice of a specific TNF antagonist could be associated with factors that influence drug persistence, such as age or disease severity. Demographic and clinical status variables were included in the multivariate regression model to minimize the effect of such possible confounders. Patient demographic variables included sex, age and a proxy variable for socio-economic status (the annual deductible for prescription cost, which was based on family annual income [30]). We used four mutually exclusive age categories: 18–30, 30–70 (reference), 70–80 and >80 years old, based on a preliminary analysis that demonstrated homogeneous persistence within each age category. Clinical status variables included the number of inpatient and outpatient encounters in the year prior to index, the duration of disease (defined as time from the first recording of diagnostic code of RA in the data to the index) and the presence and severity of comorbidities. We used Quan's algorithm for administrative databases [31] to determine the Charlson comorbidity score [32] during the year preceding the index, but excluding rheumatic diseases. The regression model also contained three variables for other antirheumatic (synthetic) drugs. Two binary variables were: (1) concomitant methotrexate in the 200 days prior to index, where the dispensing period of 200 days reflected average plus two standard deviations of between-dispensing intervals of methotrexate in the study cohort; and (2) dispensing of any nonsteroidal anti-inflammatory drug (NSAID) in the year preceding the index. The third drug variable was the number of previous antirheumatic drugs in the pre-study period. Dispensing records for nine drugs were sought: methotrexate, hydroxychloroquine, sulfasalazine, azathioprine, cyclosporine, minocycline, penicillamine, sodium aurothiomalate, prednisone and intra-articular triamcinolone/methylprednisolone acetate. We included a categorical variable for the calendar year of the index date, which allowed adjustment for possible secular trends in clinical practice [33], [34] and late availability of adalimumab (3 years after infliximab and etanercept).

Finally, we included a series of 12 binary variables for high-volume prescribers who initiated more than 70 courses in the study cohort. This series allowed adjustment for differences (an increase or a decrease) in the risk of discontinuing any TNF antagonist in patients treated by a particular physician. We used the prescriber recorded on the first dispensing claim for TNF antagonist as a proxy of the care-providing physician. The prescribers were determined using an anonymized identifier.

Our outcome was persistence (continuous variable), measured in years from index until drug discontinuation. Drug discontinuation was defined as either switching to another ‘biologic’ antirheumatic drug (TNF antagonists including certolizumab and golimumab, anakinra, rituximab and abatacept) or elapsing of a drug-free interval of 180 days. A drug-free interval was defined as a period without additional dispensing of the same pharmaceutical component after the days-supply of the latest dispensing claim was exhausted. Adding-on a synthetic antirheumatic drug was not considered discontinuation. We selected a long drug free interval that was previously used in similar studies [35] to ensure that we capture events of discontinuation and not only temporary decrease in adherence (patients who had short gaps in treatment and then resume the same drug). Discontinuation date was set to the end of days-supply of the latest dispensing event or the date of the first dispensing claim of a different ‘biologic’ antirheumatic drug, whichever was earliest. Patients were considered censored if they were continuously treated with the first TNF antagonist on December 31, 2009 (end of follow-up period) or during an interruption of more than six days in the provincial MSP coverage. The most common causes of prolonged or infinite interruptions were emigration from the province and death.

Original data on the number of days-supply recorded in PharmaNet were unreliable and frequently did not match the quantity dispensed. Therefore we also calculated days-supply based on dispensed quantity, which, if required, was imputed using the recorded total cost. We considered the prescription cost to be the most accurate and reliable quantity field; given the high cost of these medications, the number of vials or milligrams dispensed could be easily defined. We used the longest duration of days-supply, recorded or calculated, to determine both the length of a drug-free interval and the date of drug discontinuation.

### Statistical Methods

Baseline characteristics were presented and compared across the three drug groups. We assumed normality of continuous variables and the significance of differences between groups was assessed with one-way analysis of variance (ANOVA) F-test for continuous variables and the Pearson's Chi-square test for categorical variables. The crude and adjusted hazard ratios for drug discontinuation were estimated using Cox proportional hazards regression. Three pairwise comparisons were presented: infliximab versus adalimumab, infliximab versus etanercept and adalimumab versus etanercept. Drug-sex and drug-age interactions were examined but were included in the final models only if the overall significance was <0.1. We also presented the adjusted hazard ratios for all variables included in the model. We tested linearity of continuous variables and categorized non-linear variables. We also tested model assumptions including the proportional hazards and absence of interactions and found them to be valid. All calculations were performed using the SAS software package Version 9.1 (SAS Institute Inc., Cary, NC).

## Results

**Figure 1:**
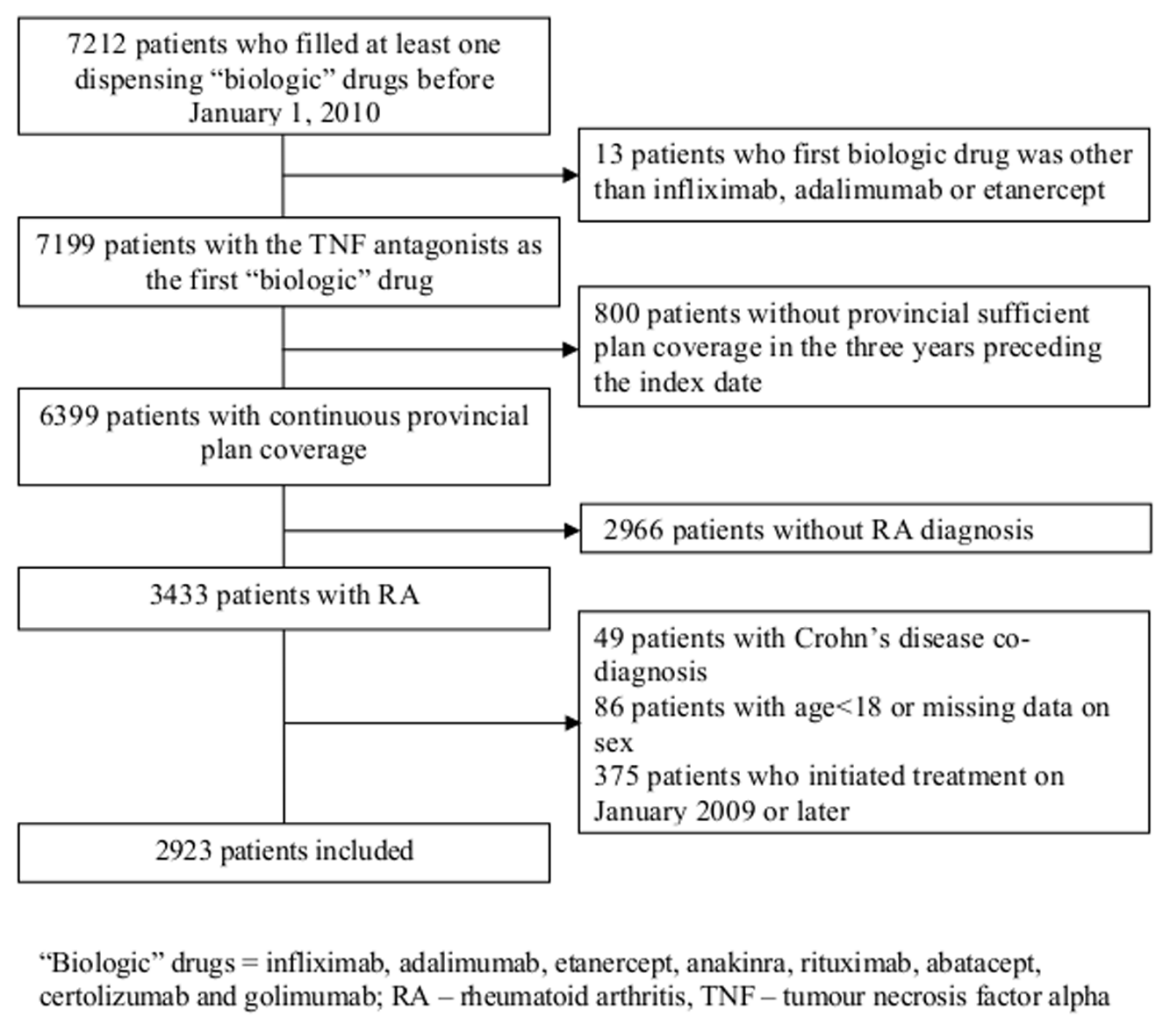
Patients' Flow.

The study cohort included 2,923 RA patients who initiated TNF antagonists by December 31, 2008 and met all selection criteria (Figure 1). The cohort was predominantly women (72%) and patients treated with etanercept (63%) (Table 1). Ages ranged from 18–92 years (median 56). Patients treated with infliximab had the highest prevalence of concomitant methotrexate therapy and patients with etanercept the lowest. This reflects differences in the indications mentioned in the product monographs; while infliximab is indicated for use in combination with methotrexate [36], the monograph of etanercept mentioned that it “can be initiated in combination with methotrexate … or used alone” [37]. Similarly, while the provincial special authority policy requires that infliximab is used in combination with methotrexate (or other drug), such requirement does not exist for treatment with etanercept or adalimumab [29].

**Table 1:** Baseline Characteristics of Patients.

Factor	Infliximab (n = 620, 21%)	Adalimumab (N = 474, 16%)	Etanercept (n = 1829, 63%)
Females, n (%)	438 (71)	344 (73)	1322 (72)
Age	56 (18–87)	58 (22–91)	56 (18–92)
Low deductible ($1–500), n (%)	34 (5)	56 (12)	162 (9)
Medium deductible ($501–2250) n (%)	96 (15)	118 (25)	356 (19)
High deductible (>$2250) n (%)	40 (6)	46 (10)	169 (9)
No deductible n (%)	49 (8)	83 (18)	213 (12)
Number of outpatient visits	33 (3–158)	31 (2–112)	32 (3–136)
Number of admissions	0 (0–6)	0 (0–5)	0 (0–8)
Number of comorbidity	0 (0–6)	0 (0–8)	0 (0–7)
RA duration in years	9.1 (0.1–17.9)	7.7 (0.3–17.9)	8.2 (0–17.8)
MTX users n (%)	417 (67)	277 (59)	902 (49)
NSAID users n (%)	332 (54)	224 (47)	983 (54)
Number of previous drugs	4 (0–8)	4 (0–8)	4 (0–9)
Calendar year	2003 (2001–2008)	2007 (2004–2008)	2005 (2001–2008)

Values are median (range) unless otherwise specified. Please refer to text (Patients and methods) for further details on the variables.

%- percent; MTX- methotrexate; NSAID- nonsteroidal anti-inflammatory drug; RA – rheumatoid arthritis; $ Canadian dollars.

**Figure 2:**
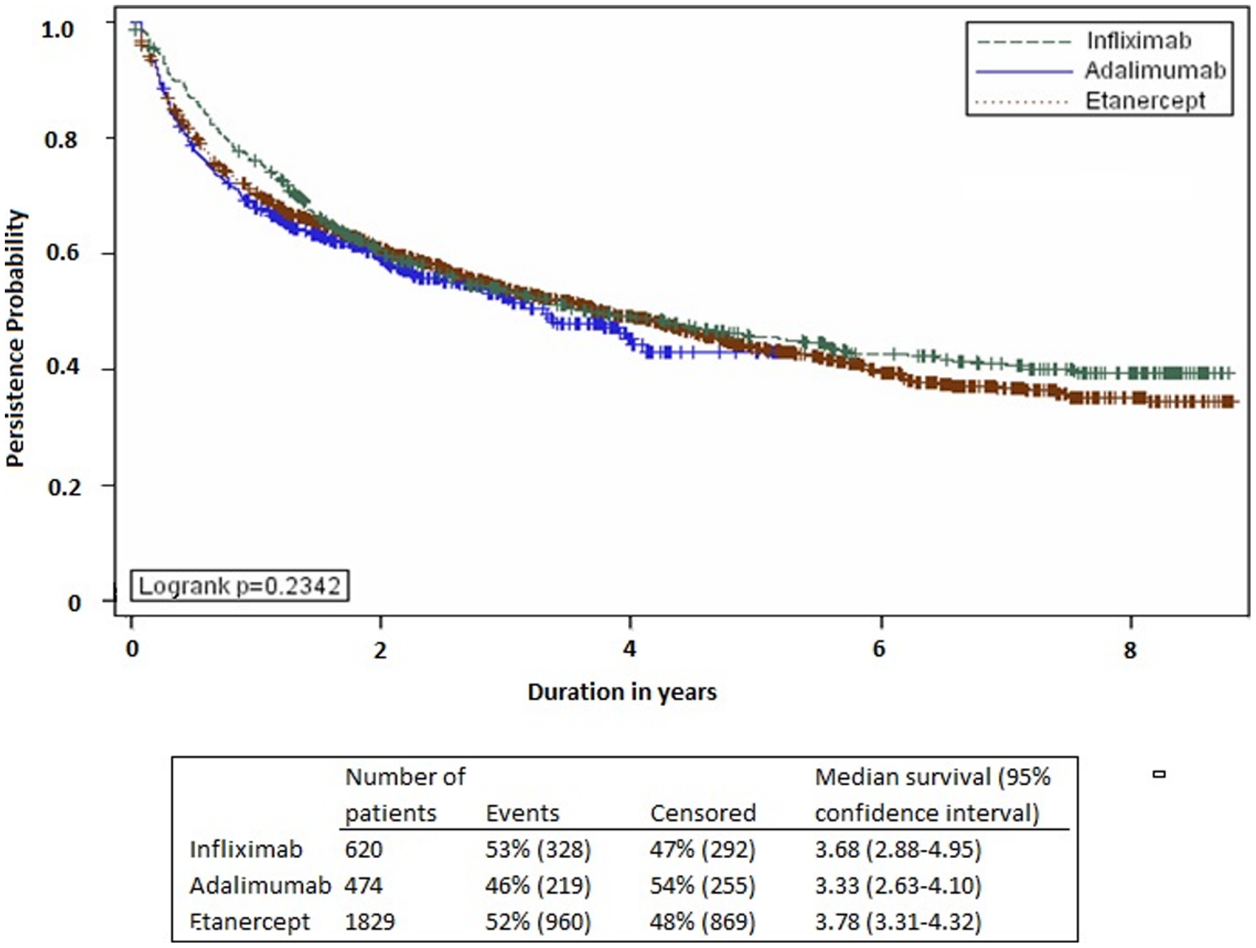
Drug Persistence Curves.

The three TNF antagonist drugs had similar persistence (median roughly 3.5 years, Figure 2). In the multivariable analysis, the estimated adjusted hazards were comparable for the three TNF antagonists (Table 2). Drug-sex and drug-age interactions were insignificant and were not included in the final model.

**Table 2:** Hazard Ratios for Discontinuation of Tumor Necrosis Factor Alpha Antagonists.

Comparison	Crude hazard ratio (95% CI)	Adjusted hazard ratio (95% CI)
Infliximab versus etanercept	0.92 (0.81–1.04)	0.98 (0.85–1.13)
Infliximab versus adalimumab	0.87 (0.73–1.03)	0.95 (0.78–1.15)
Adalimumab versus etanercept	1.06 (0.92–1.23)	1.04 (0.88–1.22)

CI – confidence interval.

The following patients' subgroups had a decreased discontinuation hazard (improved persistence): males, age 30–70, patients without admission to hospital in the year preceding treatment initiation, patients on concomitant MTX or NSAIDs treatment and patients previously treated with less than 7 antirheumatic drugs (Table 3).

**Table 3:** Predictors of Discontinuation of Tumor Necrosis Factor Alpha Antagonists.

Factor (reference)	P value (log likelihood test)	Adjusted hazard ratio (95% CI)
Sex (female)	<0.0001	0.76 (0.68–0.86)
Age (30–69 years)	<0.0001	18–29 years 1.53 (1.20–1.96); 70–79 years 1.32 (1.14–1.54); ≥80 years 1.83 (1.39–2.41
Deductible	0.8	
Outpatient visits	0.051	
Admissions (no admission)	0.006	1 admission 1.26 (1.09–1.47); 2 admissions 1.10 (0.86–1.42); >2 admissions 1.51 (1.09–2.10)
Comorbidity	0.23	
RA duration	0.07	
MTX use	<0.0001	0.79 (0.71–0.88)
NSAIDs use	0.03	0.89 (0.80–0.99)
Previous drugs (3–6 RA drugs)	0.03	No drug 1.38 (0.99–1.92); 1–2 drugs 1.04 (0.90–1.20); 7–9 drugs 1.40 (1.09–1.81)
Index year (categories)	0.08	
Prescriber-specific risk (a group of prescribers with <70 courses)	<0.0001	Physician 0.57 (0.42–0.76); physician 0.67 (0.47–0.98); physician 1.38 (1.03–1.84); physician 1.57 (1.26–1.96)

Hazard ratios are presented only for factors that are significant predictors of persistence (p-value of log likelihood test ≤0.05) and for 4 prescribers with hazard ratios that are significantly different than the reference.

CI – confidence interval, MTX – methotrexate; NSAID – nonsteroidal anti-inflammatory drug, RA – rheumatoid arthritis.

We demonstrated variation in prescribing practice between the physicians, expressed as differences in the risk for discontinuing any TNF antagonist in patients treated by different physicians (Table 3). Physician-specific risk was estimated through a series of binary variables for each of 12 heavy-volume prescribers who initiated more than 70 courses in the study cohort. Each binary variable represented deviation in ‘baseline risk of discontinuing’ for patients treated by a particular prescriber. We found that patients treated by two ‘heavy-volume’ prescribers had an increased risk of discontinuation (hazard ratios significantly higher than 1) and patients treated by another two had a significantly lower risk (hazard ratios significantly lower than 1) (Table 3) compared to patients treated with low to medium-volume prescribers. This physician-specific baseline risk of discontinuation was a significant predictor of persistence (p-value of log likelihood test <0.0001). None of the remaining covariates were associated with persistence.

## Discussion

This study found no significant difference in persistence between infliximab, adalimumab and etanercept prescribed for RA patients in British Columbia despite known differences in their pharmacological properties. The theory that treatment persistence can serve as a measurable proxy for real-world therapeutic benefit and harm implies similar long-term benefit and harm of the three drugs in RA patients. In the absence of any safety concerns that are specific to a particular TNF antagonist, it would be reasonable to base the TNF antagonist drug formulary, at least in part, on non-therapeutic factors such as cost, convenience and availability. Even if persistence is not a suitable proxy for harm-benefit in these patients, we showed that differences in convenience of administration - intravenous infusion of infliximab compared with subcutaneous injection of etanercept and adalimumab - were not associated with differences in persistence and therefore should not be considered when selecting a treatment.

Three alternative explanations are suggested for the finding of similar persistence with the three drugs. First, similar persistence could reflect similarities in the benefits and harms of the three drugs despite their different pharmacological properties. This explanation is based on the assumption that not all pharmacological properties strongly influence therapeutic effects, and the differences in these properties between the drugs would therefore not influence the clinical outcome of these drugs.

Second, similar persistence amongst the three TNF antagonists may result from benefit and harm differences in different directions. For example, some properties of infliximab might cause increased benefits compared to etanercept (e.g. apoptosis of activated T cells and circulating monocytes [38] or lack of binding of lymphotoxin alpha. [39], [40]). Potentially cancelling the increased benefit is the higher immunogenicity of infliximab which could lead to harm such as an infusion reaction [41] or the wide fluctuations in serum concentration of infliximab [39] which could increase the risk of exceeding the maximum tolerated concentration (leading to harm), or reaching suboptimal concentrations (leading to lack of therapeutic benefit).

Alternatively, persistence may be influenced by factors unrelated to therapeutic benefit or harm and the influence of these factors might be different between the drugs. For example, infliximab requires intravenous administration by health care professionals while both adalimumab and etanercept are self-injectable (subcutaneous administration). Patients treated with infliximab may discontinue treatment due to patient or physician preference for subcutaneous administered drugs [42]. This negative influence on persistence due to intravenous administration may be counter balanced by regular physician follow-up that has been shown to improve adherence and persistence to drug therapy in a variety of diseases [43], [44].

Similar persistence with the infliximab, adalimumab and etanercept has previously been demonstrated in some studies [45]–[48], while differences in persistence was observed in others [18], [19], [49]–[55]. Controversial results of previous studies may be due to different population or methodology (e.g. crude versus adjusted estimates). An in-depth systematic exploration of the reasons for the differences between the studies is beyond the scope of current study and requires further research.

The two main strengths of our study are: the characteristics of the data used and accounting for differences in practice between physicians. The universal nature of the Canadian health care system minimized selection bias and thus increased the external validity of our results. Patients were included regardless of age, employment status, socioeconomic status and heath facility use, all factors that could potentially caused biases in previous research that used administrative data, clinical chart review, or registries. Furthermore, the British Columbia Ministry of Health uses a largely systematic and standardized approach to data collection, which ensures the better representativeness of real life treatment patterns and drug-taking behavior. Access to complete information on all dispensed prescription drugs and multiple sources of data (physician encounters, hospital separation and pharmacies) contributed to the usefulness of the data. The data also granted a large sample (N = 2923), which provided ample power, as well as relatively long durations of follow up; roughly nine years.

We accounted for differences in prescribing practice between physicians by adjusting for the baseline risk of discontinuing any TNF antagonist in patients treated by different physicians. Differences in prescribing habits could be caused by differences in knowledge or adherence to different guidelines. Alternatively, patients treated by the same physician may be more similar than patients treated by different physicians in parameters such as geography, socioeconomic status, ethnic background, or age [56], and that could cause differences in the baseline risk of discontinuation. Confirming our finding, Zhang et al 2011 [57] demonstrated differences in physician response to decreased benefit with TNF antagonist therapy.

Several limitations are apparent in in analysis of administrative health data and are caused by absence of clinical information. We were unable to identify reasons for drug discontinuation in our cohort. However, based on previous studies, we assumed that the most common reasons were decreased benefit or increased harm. We also did not have access to possible clinical confounders, and therefore used proxies. This may result in residual confounding that has the potential to bias the results.

Diagnostic accuracy might be compromised or biased because disease diagnoses are coded and recorded in the databases for payment purpose. In addition, differences in accuracy of codes between the care-providers (e.g., different physicians) and variation in coding precision in different diseases are to be expected. To minimize the effect of these sources of inaccuracy, and in the absence of validated algorithm appropriate for our data, we designed a disease algorithm requiring at least two outpatient encounters with RA diagnosis.

We identified errors in recording days-supply in our data, which may be the result of refill policy of the provincial drug-coverage plan, different interpretations of days-supply, or dose titration. To overcome these inaccuracies we used a conservative estimate of days-supply (the longest among several possible approaches to estimation) that might have led to underestimation of the actual length of drug-free intervals, false late ascertainment of drug discontinuation and overestimation of drug persistence. Dispensing data for the three drugs were treated similarly to ensure that the relative hazard ratios were not systematically biased in this regard.

Inaccuracies in recording days-supply also limited our ability to identify patients with dose adjustment. Two analyses of administrative databases [48], [55] previously evaluated the frequency of dose escalation in RA patients treated with TNF antagonists showed that dose escalating was more common in patients treated with infliximab. The limited ability to identify dose adjustment in our study may have resulted in overestimation of the days-supply for patients who experienced dose escalation, mainly patients treated with infliximab, and biased estimated comparative persistence of infliximab versus adalimumab or etanercept.

We used a long drug-free interval to ascertain discontinuation rather than direct recording of discontinuation (e.g., medical records or patient reporting). This long drug-free interval algorithm minimized the number of patients considered discontinuers for a temporary interruption. We claim that temporary interruptions should be considered noncompliance, not discontinuation. By using short drug-free intervals, previous studies captured a complex measure of both persistence and compliance and therefore the end point in these studies should not be considered discontinuation. However, by using a long drug-free interval, our analysis was more vulnerable to immortal person-time bias. Patients were required to be alive at the end of drug-free interval to be considered discontinuers; otherwise, their data was censored. Patients who died due to drug effects (harm) were not considered discontinuers but their follow-up time was accounted for. This bias might have affected our comparative estimates if death related to drug effects was significantly more frequent with one of the drug, a condition we did not expected to exist.

The study results indicate that persistence with infliximab, adalimumab and etanercept is similar in RA patients – a finding that does not disapproves the hypothesis that the drugs' benefit and harm could be considered equivalent. Accordingly, clinical and policy decisions for treatment with TNF antagonist could be based on convenience and cost. Even if persistence is not a suitable proxy for harm-benefit profile, our results show that persistence should not be a consideration when selecting a TNF antagonist in RA patients.
